# Designing Scientific Advisory Committees for a Complex World

**DOI:** 10.1002/gch2.201800075

**Published:** 2018-09-27

**Authors:** Steven J. Hoffman, Trygve Ottersen, Prativa Baral, Patrick Fafard

**Affiliations:** ^1^ Global Strategy Lab York University/University of Ottawa Ottawa Canada; ^2^ Dahdaleh Institute for Global Health Research Faculty of Health and Osgoode Hall Law School York University Toronto Ontario Canada; ^3^ Department of Health Research Methods Evidence & Impact and McMaster Health Forum McMaster University Hamilton Ontario Canada; ^4^ Department of Global Health & Population Harvard T.H. Chan School of Public Health Harvard University Boston MA USA; ^5^ Division for Health Services Norwegian Institute of Public Health Oslo Norway; ^6^ Department of Community Medicine and Global Health and Centre for Global Health Institute of Health and Society University of Oslo Oslo Norway; ^7^ Graduate School of Public and International Affairs University of Ottawa Ottawa Ontario Canada

Policymakers and researchers alike have called for a greater focus on evidence‐informed decision making.^1^, ^2^ For decisions to be truly informed by scientific evidence, decision‐makers must continuously seek scientific advice as part of a well‐functioning policy advisory system.^3^ Scientific advisory committees (SACs) are often a critical part of this process, and offer the potential of systematically identifying and assessing policy options in light of the best available scientific evidence.^4^, ^5^ New committees are constantly being created and old ones reformed worldwide.^4^, ^6^ In fact, many countries routinely rely on expert panels of various kinds to inform public policy. Yet, there is surprisingly little scholarly discussion of the process of science advice and, in particular, the institutional design features that influence the operations of SACs and what makes these committees effective. The result is that existing and new SACs may not be operating as effectively as they could, meaning that policy and program choices may not be as well informed by the best available research evidence as possible.

The articles in this special issue of *Global Challenges* on the institutional design of SACs bring together a broad suite of insights from researchers across several disciplines, including public health, medicine, economics, history, law, and political science.^7^, ^8^, ^9^, ^10^, ^11^, ^12^, ^13^, ^14^, ^15^, ^16^, ^17^ The articles offer differing perspectives on what constitutes an effective SAC and what factors make SACs more effective. Read together, the special issue offers a rich array of ideas and options for ensuring the optimal design and operations of SACs which, in turn, increases the chances that decisions are informed by the best available research evidence.

This series comes at a fruitful time. In the current global political climate, it sometimes seems that policy decisions are made solely on the basis of short‐term partisan or ideological concerns with little or no consideration given to the relevant scientific evidence. This is the result, in part, of decisions by some national governments to quietly alter the membership of numerous SACs or outright dissolve them without warning. Many of these committees, initially formed to advise various government policymakers on a wide array of pressing issues, have either been sidelined or their membership changed to give conflicted or partisan representatives a much stronger voice.^18^ More generally, there is a growing mistrust of ‘experts’ sometimes linked to the rise of populist political parties of various kinds. Some have even suggested we live in a post‐truth world.^19^ In the face of these troubling trends, it is our hope that this special issue offers insights into how to optimally design SACs to ultimately bring the best‐available research evidence to bear on complex policy decisions.
